# Building a Natural Language Processing Augmented Information Support System to Enhance Supportive Care for Patients With Prostate Cancer and Families: User-Centered, Iterative Approach

**DOI:** 10.2196/74150

**Published:** 2026-08-10

**Authors:** Lixin Song, Xiaomeng Wang, Fei Yu, Dongmei Zuo, Lisa Hart Ranzinger, Michael Liss, Hung-Jui Tan

**Affiliations:** 1 School of Nursing, The University of Texas Health Science Center at San Antonio, 7703 Floyd Curl Drive, San Antonio, TX, United States, 1 210 567 5824; 2 School of Information and Library Science, University of North Carolina at Chapel Hill, Chapel Hill, NC, United States; 3 School of Nursing, University of North Carolina at Chapel Hill, Chapel Hill, NC, United States; 4 University of California, San Diego, San Diego, CA, United States; 5 Department of Urology, University of North Carolina at Chapel Hill, Chapel Hill, NC, United States

**Keywords:** prostate cancer, health information, social support, eHealth, digital health, family caregiver, self-management, decision making, user-centered design, natural language processing

## Abstract

**Background:**

Patients with prostate cancer and their families face significant challenges during transitions from diagnosis to treatment and posttreatment self-management, particularly in accessing, understanding, and using complex health information.

**Objective:**

We aimed to develop the Interactive Prostate Cancer Information, Communication, and Support Program (iPICS), a natural language processing (NLP)–augmented eHealth platform designed to enhance care continuity, support decision-making, and improve health outcomes for patients and families.

**Methods:**

This study used an iterative, user-centered design approach to design, develop, and refine iPICS, guided by responsible AI principles. The iterative development process advanced from an initial needs assessment through iterative formative and summative prototype evaluations, culminating in a final evaluation of field deployment readiness via semistructured interviews and focus groups with patients with prostate cancer and family members from diverse sociodemographic backgrounds. Thematic analysis was conducted to identify critical functionalities and content, using double coding and team-based consensus procedures. Participants’ feedback was integrated during the platform’s refinement to ensure iPICS’ usability, accessibility, security, and functionality.

**Results:**

A total of 18 patients with prostate cancer and 7 family members participated in 2 semistructured interviews and 17 focus groups. Most participants were older adults and had at least a high school education. Participants identified 5 major themes relevant to iPICS development: functional requirements, user interface design recommendations, content and visualization needs, program delivery preferences, and privacy and data security concerns. These themes informed the iPICS prototype design, development, and refinement, which include 3 core components: Inform, a multimedia health information resource hub; Dialog, an NLP-augmented consultation recording summarization tool for patient-provider communication; and Snap, a moderated online peer-support forum. Key features of iPICS include: Inform is an evidence-based, guideline-informed multimedia health information paired with National Institutes of Health–sponsored MedlinePlus papers; Dialog is an NLP-powered recording with keyword extraction and hyperlinking; and Snap is peer-support functionalities moderated by nurses to ensure safety and reliability. iPICS development was also compliant with HIPAA (Health Insurance Portability and Accountability Act) standards and aligned with user needs while ensuring usability throughout deployment.

**Conclusions:**

The NLP-augmented iPICS was developed using an iterative user-centered design approach to support patients with prostate cancer and their families. It offers a scalable solution for ethical, transparent, and inclusive supportive survivorship care, particularly during critical care transitions. As a formative qualitative study with a small sample, this phase did not evaluate patient- or family-reported outcomes. Our ongoing studies will evaluate the effects of iPICS on patient- and family-reported outcomes and explore adaptation to other cancers and chronic conditions.

## Introduction

Prostate cancer is the most prevalent malignancy among men in the United States and other resource-rich countries worldwide, excluding skin cancers [1]. Treatment decision-making and survivorship care for localized prostate cancer (LPC) present significant challenges due to the complexity of available options, including active surveillance, prostatectomy, and radiation therapy, which offer similar survival outcomes but vary in complications and side effects that profoundly impact patients’ quality of life (QOL) [2]. Thus, LPC and its treatment are often associated with uncertainty [3], anxiety [4], and decision regret [5]. Men and their families face significant psychosocial and support needs but often have limited access to personalized health information and supportive care resources [6].

Beyond initial treatment decisions, men with LPC often live with persistent urinary, sexual, and bowel problems, fatigue, and emotional distress that shape their day-to-day life and create ongoing informational and supportive care needs throughout survivorship [7]. Recent work on digital and nurse-led survivorship programs suggests that interventions incorporating tailored self-management advice are generally feasible and acceptable, although their effects on longer-term outcomes such as patient activation and health-related QOL remain mixed [8,9]. Family caregivers, who frequently coordinate care, manage symptoms at home, and provide emotional and practical support, also describe feeling underprepared for their role and report substantial unmet informational and psychosocial needs after treatment completion [10,11].

Although information seeking and processing are critical for informed decision-making and improved health outcomes, men with LPC, with a median age of a cancer diagnosis at 67 years, often struggle to find, retrieve, and retain complex medical information due to a decline in cognitive and sensory functions, particularly during medical encounters. Research has shown that 40%‐80% of information provided during medical consultations is forgotten almost immediately, with recall often incomplete and erroneous due to factors such as emotional stress, low health literacy, and the sheer volume of information presented [12]. This challenge is particularly acute during diagnosis and posttreatment care transitions, as patients and families must manage complex treatment decisions, emotional and cognitive burden, overwhelming information, and major changes in daily functioning.

Randomized and comparative studies suggest that online decision aids may improve aspects of the consultation process and patient-clinician communication and can reduce decisional conflict, even when gains in knowledge or downstream decisional outcomes are modest [13,14]. At the same time, emerging work indicates that survivors of prostate cancer and their partners often have limited eHealth literacy and may struggle to locate, appraise, and use online information effectively, which may constrain the benefits of existing digital resources [15,16].

In parallel, AI and, in particular, natural language processing (NLP) are increasingly used in oncology to extract clinically relevant information from electronic health records, pathology and radiology reports, and clinical notes, and to support tasks such as cancer registry case finding, trial matching, and symptom monitoring [17,18]. However, most current NLP applications are designed for clinicians, researchers, or health system operations rather than to directly help patients and family caregivers access, process, and retain information from real-world clinical encounters.

To address these challenges, guided by stakeholder input, the best practices in user-centered design [19], the current study aims to (1) identify critical functionalities and content to deliver personalized, reliable information, enhance patient-provider communication, and facilitate peer support; and (2) integrate user recommendations and preferences into the design, development, and refinement of a personalized information delivery system to optimize user experiences. The development of the NLP and machine learning components of the Interactive Prostate Cancer Information, Communication, and Support Program (iPICS) was guided by the established responsible AI principles [20,21].

## Methods

### Study Design

This multisite qualitative study uses an iterative, user-centered design incorporating qualitative interviews and focus groups. We used a constructivist methodological orientation informed by Braun and Clarke’s thematic analysis framework [22], which supported inductive coding and iterative theme development rooted in participant perspectives and user-experience data.

This study is the phase I report of a 2-phased mixed-methods project that aimed at developing and testing an NLP-augmented information system to support care continuity for patients with prostate cancer and their families. Evaluation of the intervention’s impact on clinical outcomes is ongoing and will be presented in a future manuscript.

### Ethical Considerations

This study was reviewed and approved by the Institutional Review Board (IRB) of the University of North Carolina at Chapel Hill (IRB#: 21‐0785) and the University of Texas Health Science Center at San Antonio (IRB#22‐0724). Conducted in accordance with human participants’ research regulations, all patients and family members provided written or IRB-approved verbal informed consent before participating in interviews and focus groups. All participants were informed that recording was voluntary, could be stopped at any time, and would not affect clinical care, and that the data could be used for research under IRB oversight.

Audio recordings were stored on secure, password-protected servers and transcribed with direct identifiers removed; deidentified transcripts were maintained in REDCap (Vanderbilt University) with role-based access controls, and all analyses used deidentified data. Participants received US $30 for each interview and US $30 for each focus group as a token of appreciation for their participation. All interface figures and screenshots in this manuscript were generated with test accounts and deidentified content; no images of identifiable individuals are included.

### Participants

Patients were eligible if they were diagnosed with LPC and completed treatment decision-making within the past 5 years. Patients could invite a family member (male or female intimate partner, an adult child, a close friend, etc) to participate. Neither patients nor family members must have major vision or cognitive impairment. All participants were 18 years or older and would read and speak English.

### Recruitment

Patients were recruited by clinician referrals at the University of North Carolina at Chapel Hill and the University of Texas Health Science Center at San Antonio using a purposive sampling approach based on a purposive sampling strategy to ensure relevance to this study’s objectives. Clinicians informed the research team when they identified patients who met this study’s eligibility criteria. The research team then contacted these patients and their families by phone or in person to introduce themselves, explain this study’s purpose and process, address questions, and obtain informed consent from those who agreed to participate.

### Study Procedure

#### Overview

Once consented, patients and families had the option to participate in either individual interviews or focus groups for iterative discussions based on their preferences and privacy concerns. Sessions were held in person or online using the institution’s HIPAA [Health Insurance Portability and Accountability Act]-compliant Zoom (Zoom Communications, Inc) platform and were facilitated by roles (eg, PhD-prepared nurse scientists or trained research staff), female PhD-prepared investigators (including FY), and male and female research assistants (including LHR). Interviewers and focus group moderators had experience conducting qualitative interviews and focus groups and completed study-specific training on the interview and focus group guides, ethical conduct, and consistent facilitation procedures. Interviews included 1‐2 participants, and focus groups included 3‐10 participants. Flexible scheduling was provided to accommodate participants’ availability. Initial contact with participants was established during the screening and consent process. At the beginning of each session, interviewers reviewed the purpose, and individual interviews and focus groups lasted 45‐75 minutes. Consistent with an iterative, user-centered development approach, some participants contributed feedback in more than 1 interview or focus group session across prototype development steps, depending on their availability and which component was under review. Accordingly, the counts reported within each step refer to individuals who contributed feedback during that step, whereas the number of participants per focus group reflects session attendance. For focus groups, only participants and study team members were present; no nonparticipants attended the sessions.

Recruitment and sessions were conducted by members of this study’s team (LS, FY, LHR, ML, and HJT). Participants had contact with this study’s team through clinician referral and the consent process; beyond this, no ongoing relationship was established before participation. At the start of each session, the interviewers introduced their role as researchers, explained this study’s purpose, and emphasized that there were no right or wrong answers and that feedback (including criticism) would inform system design, development, and refinement. The multidisciplinary team discussed potential biases (eg, enthusiasm for the intervention) during analysis meetings and used team-based consensus to minimize individual interpretation bias.

**Figure 1.**
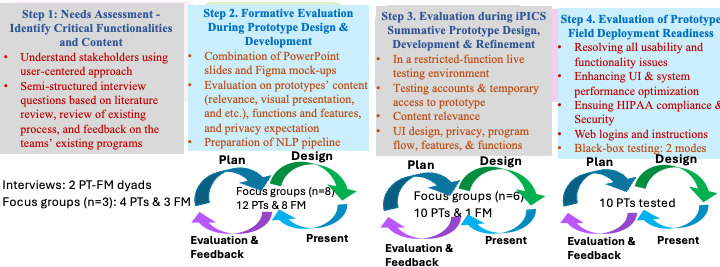
User-centered design framework and associated development steps. FM: family member; HIPAA: Health Insurance Portability and Accountability Act; iPICS: Interactive Prostate Cancer Information, Communication, and Support Program; NLP: natural language processing; PT: patient; UI: user interface.

The development process comprised four sequential steps: (1) needs assessment, (2) formative prototype evaluation, (3) summative prototype testing, and (4) prototype field deployment readiness evaluation. The prototype evaluation steps were conducted using structured, iterative cycles encompassing planning, design, presentation, and evaluation, with stakeholder feedback systematically integrated to guide ongoing refinement (Figure 1). Each step produced data-driven refinements to content, usability, relevance, and workflow integration. Using a researcher-developed interview guide with open-ended questions tailored to each step and prototype stage, interviews and focus groups were conducted across iterative cycles of prototype development and refinement, and concluded when data saturation was reached, and no new themes emerged. Saturation was determined through iterative, team-based analysis of emerging codes and themes after each cycle. As later sessions produced minimal new thematic content and largely confirmed previously identified needs and usability issues, we concluded that thematic sufficiency had been achieved for this study’s aims.

Interviewers also took field notes during the sessions, which were discussed during team meetings to inform subsequent sessions and later used to support data analysis and interpretation. Participants’ feedback and preferences gathered from earlier steps were systematically reviewed by the research team and incorporated into subsequent system design, development, and refinement cycles. This iterative process ensured the system evolved to align with user requirements and addressed participant recommendations at each stage. Given that data collection was embedded within iterative prototype refinement cycles, saturation was assessed in relation to core design themes and usability domains, rather than to comprehensively capture all experiential dimensions of LPC survivorship.

#### Step 1: Needs Assessment—Identifying Critical Functionalities and Content

Semistructured interviews and focus group sessions with patients with prostate cancer and their family members (ie, participants) were used to identify their information needs, usability expectations, and priorities for supportive care delivery. To reduce participant burden and facilitate discussion, we presented a blueprint of the iPICS, an innovative NLP-augmented eHealth intervention system. iPICS seeks to enhance information access, processing, and retention to support shared decision-making and improve QOL for males with LPC and their families, while ensuring continuity of care across diagnosis, treatment, and survivorship.

The IRB-approved questions included “Based on what you’ve just heard/seen about the system, what are your thoughts on the information it provides?” “What specific features would you like the system to provide?” “How do you think we can make the system more useful for both you and your family?” These interview questions were adapted during subsequent steps to evaluate updated iPICS prototypes and elicit participant feedback on added functionality.

#### Step 2: Formative Prototype Evaluation

Based on the findings from step 1, we developed an initial formative iPICS prototype and presented it to participants using a combination of PowerPoint (Microsoft) slides and Figma mock-ups. We conducted iterative usability evaluations to examine iPICS prototypes’ clarity, functions, features, visual presentation, and security expectations. Feedback was reviewed and synthesized to revise content organization, layout, navigation, and interactive elements, while maintaining emotional support, transparency, and privacy.

We planned to use an NLP pipeline to generate keyword-level summaries from recorded medical consultations using Amazon Comprehend (Amazon Web Services [AWS]), a HIPAA-eligible NLP service. Deidentified transcripts obtained in previous randomized clinical trials among patients across various sociodemographic backgrounds [23] underwent preprocessing (removal of identifiers, normalization, and custom filtering) before entity extraction and keyword identification. The resulting summaries were reviewed for accuracy, clarity, interpretability, and perceived usefulness during iterative usability testing and are being further evaluated in a proof-of-concept study.

#### Step 3: Summative Prototype Evaluation

The iPICS summative prototype was then developed and deployed into a platform in a restricted-function live testing environment. Before each session, the research team created testing accounts and enabled participants’ temporary access to explore the iPICS prototypes and evaluate content relevance, user interface (UI) design, functionalities, privacy, and security, and report any issues for improvement and discussion. Continuous discussions with participant stakeholders and within the research team informed refinements to iPICS features and functions, UI optimization, and navigation design, ensuring that usability and functionality aligned with participants’ feedback.

#### Step 4: Evaluation of Prototype Field Deployment Readiness

The final phase involved resolving all prototype usability and functionality issues that participants identified, enhancing the UI and system performance optimization. Security measures were also meticulously reviewed to ensure compliance with HIPAA data protection standards, particularly for handling participants’ identifiable information. The platform’s functionality was validated through Black-box testing to ensure reliability across different devices and web browsers. A detailed description of this process was published previously [24]. To develop NLP-augmented features, we applied responsible AI principles to enhance transparency, inclusivity, safety, privacy, fairness, and accountability [20,21].

### Data Analysis

All encounters were audio recorded and transcribed verbatim using the Zoom platform. The research team reviewed the transcribed data to ensure data accuracy. Data were analyzed using thematic analysis with NVivo Qualitative Data Analysis Software (version 10.2.1; Lumivero). Themes were not established a priori but inductively derived from the data using Braun and Clarke’s thematic analysis guidelines [22] to ensure credibility. Two research team members (including XW) independently coded the raw data based on system design goals, and the initial codes were grouped into broader thematic domains through iterative review, hierarchical coding, and team consensus, ensuring that themes were grounded in participant experiences rather than predefined assumptions. Preliminary themes and interpretations were summarized at research team meetings and reviewed with participants during subsequent sessions for feedback, accuracy verification, and clarification as needed. Finally, a hierarchical coding tree with parent and subcodes was developed through team discussions to systematically organize domains, iteratively refine themes, and maintain analytic transparency and rigor.

A minimum intercoder agreement of 80% was achieved [25]. Beyond intercoder agreement, we enhanced the trustworthiness and rigor of the thematic analysis through multiple validation strategies. We held regular team discussions, led by the principal investigator (LS), to review codes, themes, and coded transcripts, resolve discrepancies, and iteratively refine the codebook until full consensus was reached. We documented analytic decisions and codebook evolution. Specifically, we summarized preliminary themes and invited participants to comment on whether the interpretations reflected their experiences during subsequent sessions. We also compared thematic patterns across patient and family member transcripts and across study steps to ensure that final themes were consistent, credible, and well-grounded in the data.

## Results

### Participant Characteristics

Among 54 patients approached, a total of 18 patients with prostate cancer and 7 family members consented and participated in 2 semistructured interviews and 17 focus groups conducted throughout the iPICS program development process (Table 1). The reasons for nonparticipation included ineligibility, lack of time, lack of interest, and inability to be contacted.

**Table 1.** Participant demographics (N=25). As this qualitative, iterative study prioritized feedback for system development, only minimal participant data were collected to limit the burden associated with repeated engagement.^a^

Characteristic	Patients (n=18), n (%)	Family members (n=7), n (%)
Sex		
Male	18 (100)	0 (0)
Female	0 (0)	7 (100)
Race		
White	17 (94)	6 (86)
Black	1 (6)	1 (14)
Education		
High school or above	16 (89)	6 (86)
Less than high school	2 (11)	1 (14)
Age (years)		
>66	13 (72)	4 (57)
≤66	5 (28)	3 (43)

a Sample size (N=25) represents unique participants rather than the total number of interview or focus-group attendances. As this qualitative, iterative study prioritized repeated feedback for system development, only minimal demographic data were collected to limit participant burden.

Most participants (22/25, 88%) had at least a high school education, and 68% (17/25) were over the age of 66 years. All patients had undergone treatment for LPC. A total of 6 patients underwent prostatectomy, including 1 patient who completed radiation therapy before surgery. Further, 11 patients received radiation therapy, with 3 patients also undergoing additional treatments such as chemotherapy, hormone therapy, and single-dose radiotherapy. Additionally, 2 patients opted for active surveillance as their treatment approach.

### iPICS Design, Development, and Refinement

The results of thematic analysis revealed key emerging themes, which guided iPICS design, development, and refinement.

#### Step 1: Needs Assessment – Identifying Critical Functionalities and Content

##### Overview

From December 2021 to February 2022, we conducted interviews with 2 patient-family member dyads and facilitated 3 focus groups with 4 patients and 3 family members to explore their needs and perspectives on iPICS design and development [26]. The sample sizes for these focus groups ranged from 3 to 7 participants. Thematic analysis identified 5 main themes: functional requirements, UI design recommendations, content and visualization needs, program delivery preferences, and privacy and data security concerns. These insights shaped the formative prototype.

##### Theme 1: Functional Requirements

###### Search Function

One participant expressed interest in an efficient search tool, similar to Google, integrated within the iPICS platform. They emphasized the importance of quickly locating information, such as details about symptoms, treatment options, or coping strategies, without navigating through extensive menus or unrelated content. This capability would save time, provide users with direct access to the exact information they need, and foster a sense of reliable support, particularly during moments of urgency or when users require clarity on specific topics.

*A thing that would be a search tool similar to Google, but it’s within your system…*

###### Recording

Many participants identified the ability to record conversations with health care providers as an important feature. Participants often felt overwhelmed by the information presented during medical appointments, making recall difficult and leading to frustration when key details were later missed or misunderstood.

*We both went to the appointments, but we heard different things in the appointments and so we would get back in the car and one of us would say, well, so he’s supposed to do this for 12 months and the other would say I thought he said ten.*

###### Online Forum

Participants highlighted how connecting with others in similar situations provided practical help and advice during their LPC survivorship journey. They expressed a strong interest in having an online forum feature integrated into the iPICS, allowing them to easily engage with other patients and families. Participants confirmed this dedicated space would allow them to share experiences, ask questions, and foster a sense of community, making the treatment journey more supportive.

*Well, this sounds a little silly but I’ll just tell you, because it came into my mind when you asked it, one thing that helped me is a guy who was in worse condition than I was.*

*If you're on a talk to somebody here’s my number I wish I had somebody.*

*I think those functions would be helpful because again. Yeah, I know like with my wife or like women they have their issues and women talk about their issues. Men, we don’t always talk about the issues that’s going on in males. So when you’ve got somebody, like I do now, I have a friend of mine that’s in same situation, so I’ll talk to them.*

### Theme 2: UI Design Recommendation

#### Navigation

Participants provided recommendations for UI design, with an emphasis on navigation. They preferred a flowchart-style design that could present information in a clear and logical sequence, making it easier to follow. They also suggested incorporating “… some kind of navigation in it like categories and you drill down to things which is fine.”

#### Simple and Streamlined Design

They valued simplicity, preferring a brief overview with the option to explore additional details as desired.

*Make it simple. And then you know, again, if they want to read more, they'll go into it.*

### Theme 3: Content and Visualization Need

#### Emotional Support Resources

Participants reported significant emotional distress during the diagnosis and posttreatment phases, frequently describing feelings of shock, fear, and uncertainty. The limited availability of emotional support during these periods heightened their stress and left many feeling isolated and overwhelmed. Participants emphasized a strong need for iPICS to offer readily accessible emotional support resources to better assist patients and families during these critical care transition stages.

*I had a hard time dealing with it emotionally because it is scary. I mean, it’s got the word “cancer” in it.*

#### Treatment Options and Related Side Effects

Participants felt that the information they received regarding their treatment options and the potential side effects was limited compared with what they experienced. They desired for iPICS to provide clear, comprehensive explanations to help them weigh the benefits and risks of each option and better anticipate how different treatments might impact their QOL.

*Treatment is a big deal, so one wants to be informed about the pros and cons, which I never fully understood.*

*The first thing I thought about was that it will provide assistance in the choice for me.*

#### Information for Families

Family members needed to access role-specific information and expected iPICS to address their support needs directly, such as practical advice for managing caregiving tasks and mental health, to help reduce their stress and improve their overall ability to care for their loved ones.

*It was something that was still a question for me, even though it wasn’t for him. It’s a place where the caregiver can go for information.*

*What I liked was that it was a place that I could go to look for things when (husband) was not in the mood to talk about it.*

#### Information Delivery Modes

Participants preferred multimedia for information delivery, including text-based information for self-pacing and video content for engagement and comprehension.

*I would say you’d want to see more. I’m not a person who would want to look at a whole bunch of graphs, wouldn’t go and look at a whole lot of printed material as it would be more or less one to look at the video even being more visual than having to do all that.*

*I personally like the reading and the visual.*

### Theme 4: Program Delivery Preferences

Participants valued accessibility and ease of use across devices. Desktop computers offered better visibility for detailed information review, while mobile phones supported frequent and convenient access. They considered a responsive, mobile-friendly design essential.

*When I was doing my research, I pretty much use my desktop.*

*I don’t walk up and sit down at the desk and use my computer maybe once a week, whereas I’m looking at things on my phone.*

### Theme 5: Privacy and Data Security Concerns

Participants highlighted the importance of safeguarding their personal information within the program and preferred clear and straightforward communication about iPICS’ privacy policies. They identified features such as encrypted data storage, secure login protocols, and transparency about how data is used and shared as essential for building trust.

*I guess security could be an issue and certainly will be for some people.*

### Step 2: Formative Prototype Evaluation

#### Overview

Findings from step 1 on stakeholders’ needs and preferences informed the development of the 3 key iPICS components: Inform, Dialog, and Snap. From April 2022 to February 2023, we conducted 8 focus group sessions to interactively evaluate formative iPICS prototypes. During this stage, 12 patients and 8 family members (unique individuals) contributed feedback. Focus groups comprised 3 to 8 participants per session, and most participants attended multiple sessions.

Themes from these sessions informed refinements to visual design, advanced features and functionality, privacy and security, and accessibility. The prototype was refined iteratively across multiple cycles to meet user needs and preferences.

**Figure 2.**
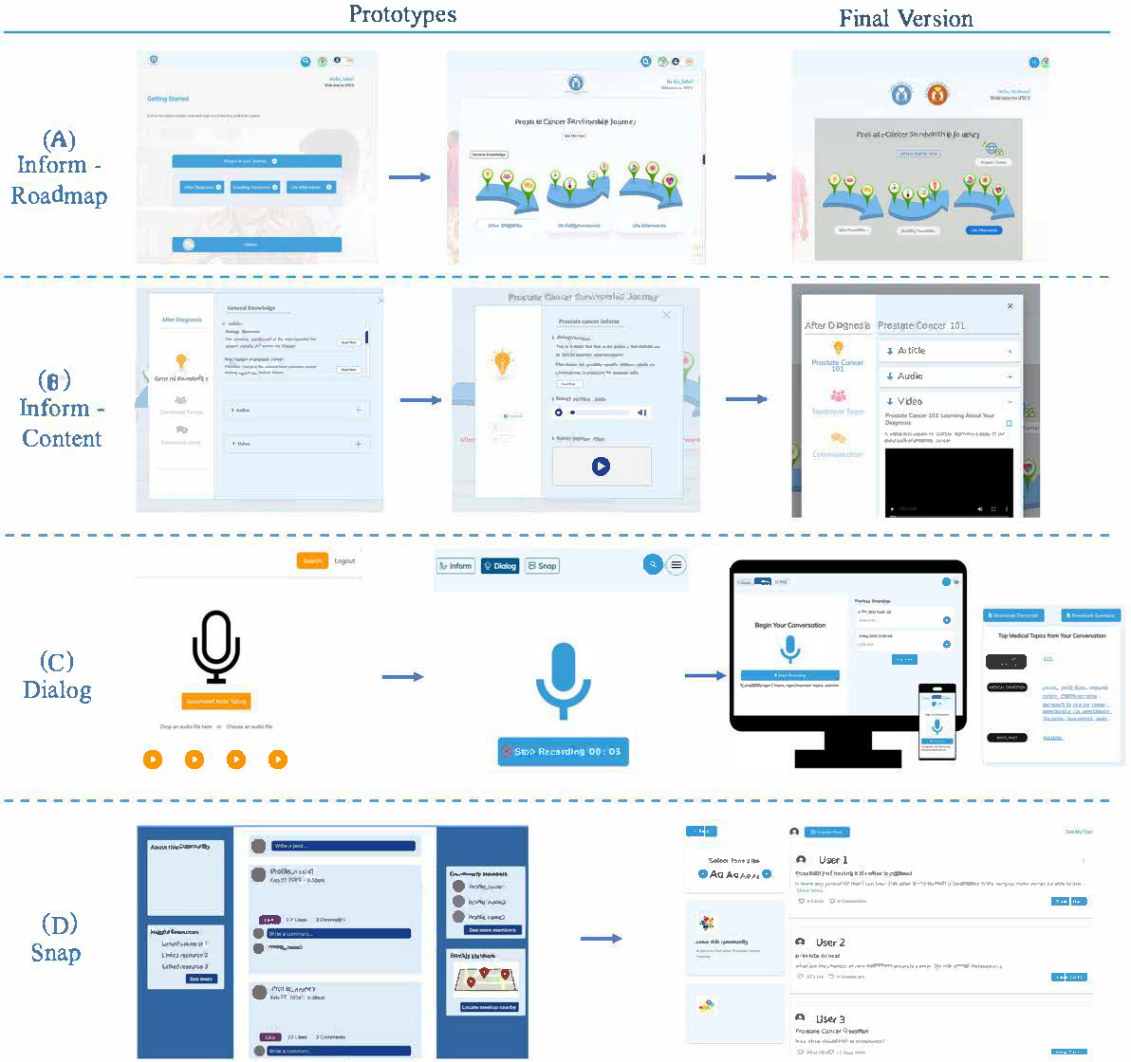
Examples of iterative design of iPICS prototypes. iPICS: Interactive Prostate Cancer Information, Communication, and Support Program.

Figure 2 demonstrates the examples of the iterative evolution of iPICS across development stages and highlights key refinements to each core component. Panel A illustrates revisions to the Inform survivorship roadmap and navigation; panel B shows how Inform content was delivered in multiple formats (text, audio, and video); panel C depicts Dialog’s recording interface and the added NLP-supported transcription, entity extraction, or summarization workflow; and panel D presents the Snap peer-support forum and its accessibility and privacy-related refinements.

#### iPICS-Inform

Although well-received with overall satisfaction, participants recommended clear cues for interactivity, “I think it’s clear, I don't think I wouldn't necessarily know that you could click on those.” The roadmap was revised to emphasize the clickable features with more visible design cues (Figure 2A). Participants subsequently reported the revised design as more engaging and functional and expressed interest in piloting it in real-world environments: “I like the roadmap screen; it looks interesting and colorful,” and “It would be easier if we could use it to figure out if it’s usable.*”*

Participants could access tailored content in the Inform section, available in text, audio, and video formats (Figure 2B), allowing them to engage in their preferred way of learning: “I like the fact that it’s presented in three different ways to learn... like our first conversation. You know, people learn differently. Learn by doing, by listening, or watching, learn by combination.”

We also incorporated a search function to enhance accessibility, enabling keyword queries across iPICS content from the website and curated MedlinePlus papers, ensuring users to quickly locate trusted and reliable health information.

#### iPICS-Dialog

The Dialog was designed as a recording system to assist users in recording their conversations with health care providers, ensuring an accurate and accessible record of important discussions during and after medical encounters. Participants acknowledged the usefulness of this function and appreciated the clarity of the design (Figure 2C).

*I think it will help the patient and the family. Particularly because the information can then be added to the family instead of just the wording from the patient. If the patient isn’t understanding everything, then there’s no way you can hear too well.*

Participants confirmed that the system should include advanced features such as text summarization to make it easier to identify key points within recorded conversations and support decision-making for patients and family members.

*That almost seems easier to have some sort of computer program that will identify, look at all this text and identify the main sections...*

To address participant needs, we enhanced the Dialog prototype to include automated voice capture, medical NLP processing, and secure summarization workflows (Figure 2C). The Dialog uses an NLP-driven pipeline starting with an AI-powered voice-to-text transcription layer that converts spoken conversations into accurate text. Amazon Transcribe Medical is used for transcription, followed by Amazon Comprehend Medical to extract prostate cancer–specific terms, medical concepts, symptoms, medications, and survivorship-related entities from the transcript. We also use a secure, HIPAA-eligible summarization layer using AWS Bedrock to generate concise, patient-friendly summaries based on both the transcript and extracted medical metadata. Custom preprocessing and filtering ensure that the final output is readable, clinically appropriate, and relevant for both patients and clinicians.

Leveraging multiple HIPAA-eligible AWS NLP services, including Amazon Transcribe Medical, Amazon Comprehend Medical, and AWS Bedrock, we have developed and refined the following features and functions: (1) an upgraded Dialog prototype capable of near-real-time audio capture and transcription; (2) automated identification of medical entities, symptoms, and prostate cancer–related concepts using Comprehend Medical; (3) large language model–powered generation of structured summaries based on transcript and extracted metadata; and (4) secure storage of voice recordings with downloadable transcripts and AI-generated summaries for review and replay.

Participants reported the summaries as helpful and easy to use and expressed enthusiasm for future testing in real-world clinical settings. Evaluation of accuracy, usefulness, and interpretability is ongoing as part of our iterative usability work and will be expanded in our proof-of-concept trial.

#### iPICS-Snap

Snap was designed as an online forum that provides a supportive space where patients and families could connect with others in similar situations, share experiences, and offer mutual support (Figure 2D).

*Hearing it firsthand from patients that, you know, been through it, had a little more weight.*

Snap aimed to foster a sense of community for patients with prostate cancer and family members.

Participants also suggested the need for a large font size to accommodate sensory declines. Additionally, they were concerned about the weekly meetups feature included in the initial prototype, worrying about the security and comfort of interacting with strangers online.

*The security of meeting somebody you don’t really know. Some folks are reluctant to do that.*

Accordingly, to protect safety and privacy, we removed the meeting feature and added professional moderation to verify users and support respectful communication.

### Step 3: Summative Prototype Evaluation

#### Overview

Between April and November 2023, we conducted 6 focus group sessions to evaluate the summative prototype. During this stage, 10 patients and 1 family member evaluated iPICS-Inform (3 sessions), Dialog (1 session), Snap (1 session), and the final refined prototype; 1 participant was lost to follow-up thereafter. Each session involved between 4 and 9 participants. Consistent with an iterative, user-centered development approach, some participants contributed feedback across multiple interviews or focus group sessions, depending on availability and prototype needs. Participant feedback on usability challenges and functionality issues informed multiple software refinements to improve user experience.

#### iPICS-Inform

The final version of iPICS Inform was developed as a dynamic resource hub to deliver personalized educational content to patients with prostate cancer and their families. The revised home page highlights the Prostate Cancer Survivorship Journey, visually represented as a guided pathway divided into 3 key phases: after diagnosis, deciding treatment, and life afterwards (Figure 2A), providing users with a clear and intuitive navigation structure. The roadmap helps users locate care phase-relevant information through interactive icons that link to tailored resources, such as emotional support materials, treatment options, side effects, and coping strategies. Participants helped refine the phase labels and reported high satisfaction with the design, highlighting its usability and convenience.

*I’m very impressed with it. I think it’s going to be very, very helpful …You have one stop shopping. I like that. And I guess my one word would be that it’s very comprehensive.*

A “helper’s corner” offered tips on family members’ self-care and guidance on supporting patients effectively. Participants chose the term helper instead of caregivers, noting the prostate cancer journey is generally less physically debilitating than many other illnesses. However, they also recognized substantial negative impacts on their loved ones and emphasized the importance of self-reliance and effective management with ongoing family support throughout the survivorship journey.

To address varied information-seeking styles and literacy levels, Inform content was developed from clinical guidelines and published scientific evidence, translated into lay language for accessibility, verified by clinicians for accuracy and reliability, and further enhanced with curated educational resources from MedlinePlus. It was delivered in multiple formats (text descriptions, audio recordings, and videos) to support diverse learning needs and ensure an engaging and accessible user experience. The videos were professionally produced in partnership with the clinician investigators. The search function was also fully integrated to enhance accessibility.

*Multiplicity of media, vocal, visual, and readable. I know it could be repetitive. But it accounts for people with other disabilities that may not be able to get a get on it or understand what’s going on.*

#### iPICS-Dialog

In the final prototype, we replaced the original red recording button with an enlarged microphone icon and a clearly labeled “start recording” button (Figure 2C) to enhance visibility and facilitate ease of use, especially for older adult users. After clicking the “start recording” button, users are prompted to confirm their consent for recording, ensuring confidentiality and verifying that all consultation participants have agreed to be recorded and the recordings are only for research purposes.

The final iPICS prototype is fully responsive and compatible with both desktop and mobile devices. We also further enhanced the performance of the keyword extraction function based on the responsible AI principles. By incorporating diverse and representative data obtained in previous randomized clinical trials among patients across various sociodemographic backgrounds [23], the function demonstrated accuracy in identifying relevant medical terminology and high-frequency terms associated with LPC. Feedback from patients, family members, and health care providers ensured alignment with domain-specific terminology and strengthened transparency through the informed consent for secure and ethical handling of sensitive health information. Participants rated the prototype as practical and useful.

*I like the keyword features. Sometimes, you know, those of us that aren’t in that profession, you hear a word, and you go, what was that again? I didn’t see it there. Maybe I missed it.*

Participants valued highlighted keywords linked to additional information for complex medical terms. We refined the NLP algorithm to extract keywords and hyperlinked them to MedlinePlus, providing users with trusted supplementary details, improving comprehension, and empowering more informed decision-making and confidence throughout the LPC journey. Patients can also easily share the recordings with authorized parties, allowing them greater control over information seeking, processing, and retention.

#### iPICS-Snap

The final prototype also incorporated the professional-moderated online forum “snap” to facilitate peer support among patients and family members. Based on our previous research [22,23] and participants’ input, we added topic themes to address the most commonly asked questions and concerns related to LPC survivorship (Figure 2D). After selecting a topic of interest, users can enter the Snap forum to participate in discussions and access posts from people who have similar backgrounds and experiences. We adjusted the color scheme to better align with the overall iPICS platform theme and changed the forum page layout from three columns to two, creating more space for post messages. Snap included a font-size selection feature to improve accessibility for older adults with vision declines, which participants tested and found satisfying.

*I like the opportunity to choose your font.*

In addition to professional moderation that mitigates misinformation, disinformation, and inappropriate content and ensures a safe, supportive environment, Snap also incorporated interactive features intended to encourage active user engagement. Users could click the “cares” button under the post to support a post and save posts for future review. A newly added comment function allowed users to leave messages and engage in discussions with others. Snap does not require profile pictures and allows users to modify their profile settings, including customizing their display name. This feature enables users’ anonymous participation, allowing users to engage in discussions without disclosing identifying personal details, such as name or location.

*I'm just curious if that would be kind of an anonymous way to let people, without sharing their locations, communicate personally.*

*I don’t want my picture out there. I mean, I don’t, and so if many people have access to this...*

### Step 4: Evaluation of Prototype Field Deployment Readiness

The final stage of the iPICS prototype development is for real-world deployment. Participant feedback guided improvements in navigation, content organization, and visual design, significantly enhancing the user experience, resulting in a more intuitive and user-friendly interface.

To ensure robust performance, iPICS was deployed on AWS, leveraging its scalability and security features. AWS Cognito was used to manage user accounts, providing secure authentication and authorization while adhering to HIPAA data protection standards. Additionally, all patient-identifiable information, for example, name, contact details, and medical-related information, was separated from the iPICS website and securely stored in an encrypted, password-protected database in REDCap, a widely trusted academic platform for managing sensitive research and clinical data. The integration of REDCap ensured data security and compliance with regulatory requirements. System performance was measured by Black-box testing to confirm the reliability and robustness of the system under diverse usage scenarios. The testing results have been published previously [24].

## Discussion

### Principal Results

This study highlights the process and importance of an iterative, user-centered approach in developing digital health interventions that are accessible, patient-centered, and responsive to the needs of diverse users. iPICS was refined through continuous input from patients, families, ensuring its 3 core components (Inform, Dialog, and Snap) effectively support survivors of prostate cancer and their families during critical care transitions. By integrating evidence-based information, NLP-enhanced recording, and moderated peer support, iPICS enhances information retrieval, decision-making, and emotional support while maintaining continuity and satisfaction with care, addressing key gaps in LPC survivorship. Furthermore, this study demonstrates the application of responsible AI principles in developing clinical support systems, emphasizing ethical design, data security, transparency, and inclusivity. Future research should focus on evaluating the real-world effects of iPICS on health outcomes and exploring its adaptability to other cancer types and chronic conditions.

Our qualitative interviews, focus groups, and thematic analysis identified the 5 key themes of user needs: functional requirements, UI design recommendations, content and visualization needs, program delivery preferences, and privacy and data security concerns. Aligning with results from previous studies [27-29], this thorough understanding of users’ needs and preferences of the critical functionalities and content provided a robust foundation for the design, development, and refinement of multiple iPICS prototype iterations. By addressing the lived experiences and expectations of its end users, iPICS demonstrates significant potential to improve the quality of prostate cancer supportive care. Similar to the challenges reported in previous research with processing and retaining complex medical information, patients with prostate cancer and family members in this study emphasized the critical need for support systems that are accessible, reliable, safe, and user-friendly. Personalized interventions such as iPICS can help empower users and alleviate these challenges, improving their overall survivorship experience.

Additionally, participant input guided the refinement of features across all components, including improving navigation tools, enhancing content clarity, and ensuring privacy through secure data management. This study also highlighted the challenges in addressing the diverse needs of users. Balancing simplicity with functionality required careful prioritization of features to maintain usability without compromising essential capabilities. Ensuring the security and ethical management of sensitive data was also a critical focus through the iterative process, with robust measures implemented to comply with HIPAA standards and foster trust among participants.

Together, these efforts enabled the team to develop the potentially transformative iPICS in bridging critical gaps in survivorship care. By addressing the informational, physical, emotional, and social needs of patients with prostate cancer and their families, iPICS offers a comprehensive, scalable solution that empowers users to navigate their cancer journey with confidence and support.

### iPICS-Inform

One common challenge reported among patients with cancer and family members is the lack of reliable and accessible information. Although patients generally perceive health care providers as skilled and empathetic, they often find health care resources to be inaccessible [30]. To address this gap, the Inform was designed to deliver reliable and accessible content tailored to the needs of patients and family members. The platform incorporates a journey map that visually guides users through the postbiopsy and posttreatment phases—stages often marked by uncertainty, stress, and long-term side effects that significantly affect QOL [31]. By addressing challenges and concerns across different stages of prostate cancer survivorship, Inform empowers users to make informed decisions and effectively manage their treatment and care during survivorship. Evidence-based brief or extensive information is available to meet the diverse information needs for patients and families during stressful times, and information delivery using multimedia such as videos, text, and audio, caters to diverse learning preferences and literacy levels, ensuring broad accessibility.

### iPICS-Dialog

Effective communication between patients and clinicians is essential for delivering high-quality care, particularly when navigating complex treatment options and survivorship pathways during stress and information overload. Research has shown that recording medical consultations improves patients’ and families’ ability to recall and retain vital health information, particularly for those aged 50 years and older [32,33]. The Dialog was developed to enhance information processing and retention of postmedical consultation. The inclusion of the recording, summarization, and hyperlinked keywords further enhances usability, easing users’ navigation of complex medical content and enabling their focus on the most relevant details. By empowering patients and families with tools to better understand, retain, and share medical information, Dialog fosters more informed and confident decision-making.

### iPICS-Snap

Online forums have increasingly become valuable platforms for patients with cancer and their families to seek support without geographic limitations [34,35]. For males living with prostate cancer and their families, these forums offer critical spaces for connecting with others who share similar experiences [36]. The snap online forum fosters a sense of community by providing a dedicated space for emotional support and the exchange of LPC survivorship experiences. Thoughtful design elements, such as topic-based discussions, customizable anonymity settings, font-size adjustments, and professional moderation, enhance usability, accessibility, and confidentiality. These features provide a secure, engaging platform where users can connect, share experiences, and support one another throughout their cancer care journey. By facilitating meaningful connections among users with shared experiences, Snap addresses the emotional and social support needs of patients with prostate cancer and their families, offering a network of mutual understanding and encouragement.

### Lessons Learned in Applying Responsible AI Principles to Clinical Support System Development

As AI technology increasingly enhances health care processes, ensuring ethical principles in AI, such as transparency, fairness, inclusivity, privacy, and security, is essential [37,38]. This study exemplifies the integration of responsible AI principles [20,21] in the development of NLP-augmented iPICS. The responsible AI principle-informed strategies outlined in Table 2 enabled the research team to develop an ethical, transparent, inclusive, secure, and accountable digital health solution for supporting patients with prostate cancer and their families.

**Table 2.** Application of responsible AI principles in iPICS^a^ Development.

Principle	Implementation in iPICS
Ethics	A multidisciplinary team ensured ethical decision-making through active participation in recruitment, interviews, data analysis, and prototype refinement. Features such as evidence-based health information (Inform) and keyword-linked consultation summaries (Dialog) empowered users to make informed decisions.
Transparency	An iterative feedback process incorporated input from patients, families, and providers at every stage. The mandatory consent process in Dialog reinforced transparency in data recording and usage. Continuous feature evaluation and refinement further ensured transparency in system design.
Fairness and inclusivity	Efforts to recruit diverse participants ensured representation across sociodemographic backgrounds. Accessibility features—multimedia content, font-size adjustments, and mobile-friendly designs—accommodated varied literacy levels and technological capabilities. Tailored resources, such as search tools and journey maps, prevented biases related to race, education, or socioeconomic status.
Privacy and security	HIPAA^b^ compliance and secure data storage on REDCap protected patient-identifiable information. Users could customize profiles for anonymity, ensuring confidentiality and safeguarding privacy.
Accountability	A multidisciplinary team led an iterative development process, ensuring alignment with user needs. The Snap forum was actively moderated to prevent misinformation, disinformation, and abusive language, maintaining a safe and supportive environment.

a iPICS: Interactive Prostate Cancer Information, Communication, and Support Program.

b HIPAA: Health Insurance Portability and Accountability Act.

### Limitations

While this study’s user-centered approach ensured that iPICS meets users’ needs and preferences, the following limitations warrant acknowledgment. First, this was a qualitative study with a relatively small sample (18 patients with prostate cancer and 7 family members) recruited from 2 academic health systems. As such, the findings are not statistically generalizable but instead provide in-depth, transferable insights into user needs and design considerations. In addition, our participants predominantly consisted of White individuals with at least a high school education, despite our efforts to recruit participants with diverse demographics, which may limit the generalizability of our findings to populations with lower levels of educational attainment. Future research should strive to expand access to a more diverse pool of patients and families to enhance representation and inclusivity in study findings. Future research should strive to expand access to patients and families with diverse backgrounds, especially those from underrepresented racial and ethnic groups, those with lower educational attainment, and those receiving care in community and safety-net settings. Offering study materials and iPICS content in additional languages and partnering with community organizations may further enhance representation and inclusivity in future studies.

Next, patients initiating recordings of medical encounters may potentially impact the trust and relationships between patients and clinicians [39] despite the existing evidence indicating that patients record their conversations with physicians [40,41]. To mitigate potential ethical issues, we designed iPICS-Dialog to include a mandatory consent process before initiating recording. Meanwhile, the physician investigators on the research team provided valuable input and reassurance regarding the recording and consenting process, helping to address potential concerns and ensure ethical and practical alignment with clinical practice. Nonetheless, future research should prioritize involving more health care providers in the study to ensure their perspectives and concerns are adequately addressed. Providing clearer guidance on the ethical and practical considerations of consultation recording will be essential for fostering acceptance and encouraging its effective use. Finally, as a phase I study, the current work focused on the design and development of iPICS and did not evaluate its effects on patient-, family-, or clinician-reported outcomes or clinical endpoints. These outcomes will be investigated and disseminated through a planned pilot study phase II and a randomized clinical trial phase III of iPICS implemented in routine oncology care.

### Implications for Future Research and Clinical Practice

#### Research Implications

Future research will formally evaluate iPICS in real-world settings, including pilot and randomized trials embedded in oncology clinics. These studies will examine iPICS’ feasibility (eg, recruitment, retention, adherence to use of Inform, Dialog, and Snap, and completion of follow-up assessments) and acceptability (eg, patient and family satisfaction with iPICS, perceived usefulness, and willingness to recommend the program). They will also examine preliminary effects on key clinical outcomes, including decision satisfaction and regret, decisional conflict, prostate cancer–specific knowledge, communication and self-management self-efficacy, emotional well-being, and health-related QOL.

In addition, process and implementation measures such as patterns of use across iPICS components, engagement with consultation recordings and NLP-generated summaries, use of the Snap forum, and qualitative feedback from patients, family caregivers, and clinicians, will clarify how and for whom the program works best and inform future refinements and adaptations to other cancers and chronic conditions.

The design and development of iPICS provide insights for developing personalized eHealth interventions aimed at improving patient-centered continuity of supportive care and shared decision-making. By leveraging NLP and integrating ongoing, diverse feedback, the platform not only addresses the immediate needs of survivors of prostate cancer and their families but also lays the groundwork for broader applications in other chronic conditions requiring complex care transitions.

#### Implications for Clinical Practice—Workflow Integration and Provider Burden

As iPICS moves toward real-world implementation, careful attention to clinical workflow integration and provider burden will be essential. Inform and Snap are intended to be used by patients and family members primarily outside of clinic encounters, enabling preparation of questions and ongoing access to educational content and peer support. This approach reduces the need for clinicians to reiterate the same information during visits; the tools, thus, lower clinician burden while ensuring that critical information needed for treatment decisions and posttreatment self-management remains accessible and uncompromised. Dialog can be integrated into existing encounters by using a brief, standardized consent process that enables patients or family members, not clinicians, to initiate and manage the recording. NLP-generated keywords and linked resources are intended to be reviewed by patients and families between visits, with optional summary views that clinicians may choose to access if helpful (eg, to tailor follow-up counseling or address unresolved concerns), rather than as a mandatory documentation step. Dialog allows patients and families to rereview recordings and summaries, reducing recall bias, ensuring shared understanding, and supporting secure information sharing. This decreases the need for clinicians to clarify or repeat information. These design choices aim to empower patients and their families in information seeking, processing, and retention, while also improving communication and continuity of care. Importantly, they achieve this without imposing additional workload or time demands on oncology teams. Our ongoing proof-of-concept trial will evaluate the feasibility and preliminary efficacy of integrating iPICS into clinical care through EPIC–MyChart and its impact on patient and clinician outcomes.

Finally, regulations governing audio recording of clinical encounters vary across states and countries, including differences between one-party vs all-party consent requirements and institution-specific policies. In this study, iPICS-Dialog was developed using consultation recordings obtained in US clinical settings where recording is permitted with consent and under IRB oversight. Physician investigators on the team also expressed strong enthusiasm for the feature and functionality. In this context, dialog requires explicit consent from both patients and clinicians before any recording. Future implementation and scale-up of iPICS will be carefully tailored to local legal and regulatory environments, as well as to clinician readiness and support, including site-specific review of applicable laws and institutional policies. In jurisdictions where routine recording is not permissible, additional safeguards or alternative approaches will be developed to ensure ethical, compliant dissemination.

### Conclusions

This phase I digital health intervention development study exemplifies the value and potential of a user-centered, iterative design in creating meaningful, patient and family-focused eHealth solutions such as iPICS to bridge gaps in survivorship care. By addressing the information, emotional, and social support needs of LPC patients and their families, iPICS represents a significant step forward in leveraging machine learning technology to enhance care continuity and clinical support during critical transitions in the cancer survivorship journey. This study also integrated responsible AI principles in developing clinical support systems, ensuring ethical, transparent, and patient-centered approaches to technology integration in health care.

## Supplementary material

10.2196/74150 Checklist 1 COREQ checklist.

## Abbreviations

AWS: Amazon Web Services
HIPAA: Health Insurance Portability and Accountability Act
iPICS: Interactive Prostate Cancer Information, Communication, and Support Program
IRB: institutional review board
LPC: localized prostate cancer
NLP: natural language processing
QOL: quality of life
UI: user interface
