# Supplementary material for: Building a Natural Language Processing Augmented Information Support System to Enhance Supportive Care for Patients With Prostate Cancer and Families: User-Centered, Iterative Approach
Source: J Med Internet Res. 2026 Aug 10;28:e74150. doi: 10.2196/74150 (PMC13456304; doi:10.2196/74150)
Supplement: Supplementary file 1 [file jmir-v28-e74150-s001.docx]

**Table S1.** Consolidated criteria for reporting qualitative studies (COREQ): 32-item checklist.

| No Item Guide questions/description | Page number |
| --- | --- |
| **Domain 1: Research team and reflexivity** |  |
| Personal Characteristics |  |
| 1. Interviewer/facilitator Which author/s conducted the interview or focus group? | 4 |
| 2. Credentials What were the researcher’s credentials? E.g. PhD, MD | 4 |
| 3. Occupation What was their occupation at the time of the study? | 4 |
| 4. Gender Was the researcher male or female? | 4 |
| 5. Experience and training What experience or training did the researcher have? | 4 |
| Relationship with participants |  |
| 6. Relationship established Was a relationship established prior to study commencement? | 4 |
| 7. Participant knowledge of the interviewer: What did the participants know about the researcher? e.g. personal goals, reasons for doing the research | 4 |
| 8. Interviewer characteristics: What characteristics were reported about the interviewer/facilitator? e.g. Bias, assumptions, reasons and interests in the research topic | 4 |
| **Domain 2: study design** |  |
| Theoretical framework |  |
| 9. Methodological orientation and Theory What methodological orientation was stated to underpin the study? e.g. grounded theory, discourse analysis, ethnography, phenomenology, content analysis | 3 |
| Participant selection |  |
| 10. Sampling How were participants selected? e.g. purposive, convenience, consecutive, snowball | 3 |
| 11. Method of approach How were participants approached? e.g. face-to-face, telephone, mail, email | 3 |
| 12. Sample size How many participants were in the study? | 6 |
| 13. Non-participation How many people refused to participate or dropped out? Reasons? | 6 |
| Setting |  |
| 14. Setting of data collection Where was the data collected? e.g. home, clinic, workplace | 4 |
| 15. Presence of non-participants Was anyone else present besides the participants and researchers? | no |
| 16. Description of sample What are the important characteristics of the sample? e.g. demographic data, date | 6 |
| Data collection |  |
| 17. Interview guide Were questions, prompts, guides provided by the authors? Was it pilot tested? | 4 |
| 18. Repeat interviews Were repeat interviews carried out? If yes, how many? | 4-5, 12 |
| 19. Audio/visual recording Did the research use audio or visual recording to collect the data? | 4 and 5 |
| 20. Field notes Were field notes made during and/or after the interview or focus group? | 4 |
| 21. Duration What was the duration of the interviews or focus group? | 4 |
| 22. Data saturation Was data saturation discussed? | 4 |
| 23. Transcripts returned Were transcripts returned to participants for comment and/or correction? | n/a |
| **Domain 3: analysis and findings** |  |
| Data analysis |  |
| 24. Number of data coders How many data coders coded the data? | 6 |
| 25. Description of the coding tree Did authors provide a description of the coding tree? | 5-6 |
| 26. Derivation of themes Were themes identified in advance or derived from the data? | 5-6 |
| 27. Software What software, if applicable, was used to manage the data? | 6 |
| 28. Participant checking Did participants provide feedback on the findings? | 6 |
| Reporting |  |
| 29. Quotations presented Were participant quotations presented to illustrate the themes / findings? Was each quotation identified? e.g. participant number | 7-14 |
| 30. Data and findings consistent Was there consistency between the data presented and the findings? | Yes 7-14 |
| 31. Clarity of major themes Were major themes clearly presented in the findings? | 7-14 |
| 32. Clarity of minor themes Is there a description of diverse cases or discussion of minor themes? | 7-14 |
